# Histological analysis of the distribution pattern of glandular tissue in normal inferior nasal turbinates

**DOI:** 10.1016/S1808-8694(15)30488-2

**Published:** 2015-10-19

**Authors:** Ieda Millas, Bianca Maria Liquidato, José Eduardo Lutaif Dolci, José Humberto Tavares Guerreiro Fregnani, José Rafael Macéa

**Affiliations:** 1 Master's degree in otorhinolaryngology, FCMSCSP. Doctoral student in otorhinolaryngology, FCMSCSP; 2 Doctorate in otorhinolaryngology, FCMSCSP. Assistant professor of the Morphology Department, FCMSCSP; 3 Doctorate in otorhinolaryngology, UNIFESP. Head of the Otorhinolaryngology Department, FCMSCSP; 4 Doctorate in health sciences, Fundação Antônio Prudente. Assistant professor of the Morphology Department, FCMSCSP; 5 Doctorate in surgery, FCMSCSP. Adjunct professor of the Morphology Department, FCMSCSP

**Keywords:** mucociliary clearance, histology, nasal mucosa, nose

## Abstract

Nasal turbinates play an important role in nasal physiology. These functions include the important function of particle filtration by the mucocilliary system. Many nasal mucosal diseases, such as rhinitis and rhinosinusitis, are directly related with structural alterations of the mucosal lining of the turbinates.

**Aim:**

To study the distribution pattern of the glandular epithelium of the lamina propria in the normal lower nasal turbinate mucosa of the anterior, medium and posterior portions.

**Material and Method:**

A prospective study in which small linear fragment of the lower nasal turbinate was removed from ten patients undergoing aesthetic nose surgery. The slides were hematoxilineosin stained, examined histologically and photographed. Glandular epithelium was delimited individually, the total area of the lamina propria on the anterior, medium and posterior portions of nasal turbinates was calculated (μm2).

**Results:**

There was no statistically significant difference in the distribution pattern of the glandular epithelium of the lamina propria.

**Conclusion:**

This study showed no predominance of glandular epithelium distribution in anterior and posterior portions of lower nasal turbinates in normal subjects.

## INTRODUCTION

The lower turbinates have an important role in nasal physiology. These are bony structures lined with mucosa (ciliated pseudostratified columnar epithelium with goblet cells) supported on richly vascular loose connective tissue, and containing many mucous glands.^1^, ^2^, ^3^ The nasal turbinates present an ample mucosal surface; this is the main functional area of the nose.^4^

Nasal turbinates protect the lower areas of the respiratory system by regulating inhaled air temperature and moisture and filtering foreign particles (with the mucociliary system).

Mucociliary clearance is one of the main functions of the nasal mucosa. Adequate clearance requires not only intact ciliary function but also sufficient production of mucus with appropriate physical and chemical properties. Goblet cells in the superficial epithelium and the glandular epithelium in the lamina propria of the nasal mucosa produce this mucus.^5^,^6^ Mucociliary clearance is essential for the airway defense system. Mucus is a physical barrier to which particles and pathogens adhere; these noxious agents are removed by the effect of cilia in the respiratory epithelium.^7^

Many nasal mucosa diseases, such as rhinitis and rhinosinusitis, are directly related with structural changes in the mucosa of the nasal turbinates. Thus, hypertrophic mucosa causes nasal block, and atrophic mucosa may manifest as a dry nose, nasal bleeding and crust formation.^8^

Changes in the physical and chemical properties of the nasal mucus alter its viscoelasticity, which in turn causes respiratory diseases. The amount of mucus should not be excessive or insufficient to respect the epithelial respiratory physiology. When the physiology of the nose is altered, diseases such as rhinitis, rhinosinusitis and lung diseases may arise.^6^

Caution is required when prescribing drugs that alter ciliary movement, decrease the production of mucus, or change its physical and chemical properties; cases in which mucus is produced excessively are also generally treated. Concerns with maintaining the homeostasis of the respiratory epithelium are constant in medical and surgical practice.

Histological and morphological studies of the lower nasal turbinates advance our knowledge on nose physiology and help us to develop therapies for treating diseases of the nasal mucosa.^8^ The area of the nasal turbinates is ample; it is the main functional site in the nose.^4^

In otorhinolaryngology, the anterior, middle and posterior segments of the nasal turbinates are named head, body and tail, although these terms are not included in the currently used Anatomical Nomenclature.^9^ The purpose of this study was to investigate the distribution pattern of the glandular epithelium in the lamina propria of the head, body and tail of normal lower nasal turbinates.

## MATERIAL AND METHOD

Ten patients (four male and six female subjects) undergoing esthetic nasal procedures were selected in by arrival order. Exclusion criteria were nasal symptoms, smokers and subjects with rhinitis and/or rhinosinusitis. Subjects consented voluntarily to the removal of a small linear fragment of the lower nasal turbinate, removed in the anterior-posterior direction, after hearing an explanation and signing a free informed consent form. The Research Ethics Committee of the Santa Casa de São Paulo approved the study (number 173/04).

Removal of a linear specimen along the full length of the medial face of the turbinate was done with turbinectomy scissors, with no prior luxation or infiltration. The width of fragments measured about 0.5 cm and the length varied depending on the nasal turbinate. Fragments were divided into head (anterior third), body (middle third) and tail (posterior third). Specimens were placed in 10% formaldehyde and separately included in paraffin blocks. A rotatory microtome was used for cutting the blocks to obtain sections with a thickness of three micrometers. These were hematoxylin-eosin stained and examined histologically.

**Figure 1:**
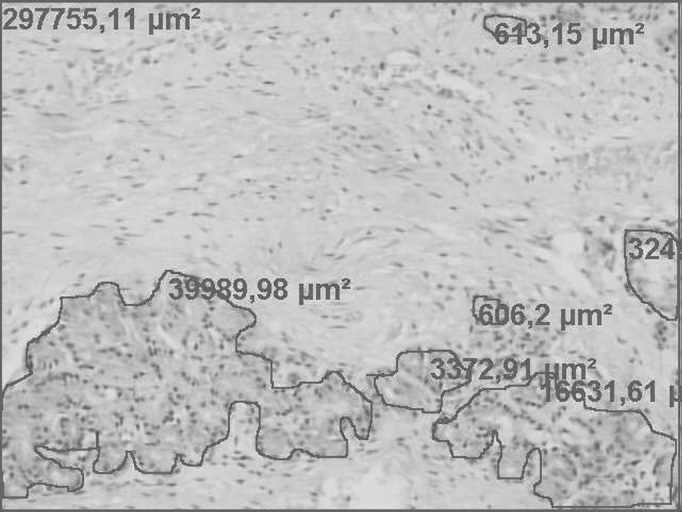
Lamina propria of the lower nasal turbinate mucosa, showing the total area and measurement of the lamina propria (red rectangle). Marked irregular and isolated areas with their measurements refer to the glandular epithelium (H&E 100X).

One field from each of the three abovementioned regions was selected for each case. Photographs were taken with a Zeiss Axioscop 40 microscope coupled to a personal computer (Intel Pentium III) and using the Axiovision 3.1 software to measure areas. In these pictures, areas of glandular epithelium and the total area of the lamina propria of the mucosa (nasal turbinate head, body and tail) were defined individually and measure in μm2 (Fig. 1).

Information was stored in an electronic database (Microsoft Office Excel 2003) and analyzed using the SPSS version 13.0 statistical package. Friedman's non-parametric test was applied for comparing the mean values of the total area of the lamina propria and the gland quotient (gland area over the total area) in the three portions of the lower turbinate. The significance level was 5%.

## RESULTS

Optic microscopy showed that the fragment had a respiratory epithelial lining – ciliated cylindrical pseudostratified with goblet cells – supported on a lamina propria with vascularized loose connective tissue and seromucous glands; the aspect was within normal limits.

There was no statistically significant difference in the distribution pattern of the glandular epithelium of the lamina propria in the head, body or tail of the lower nasal turbinates (Table 1).

**Table 1:** Mean values of gland area, the total area of the lamina propria and the gland quotient in the head, body and tail of the lower nasal turbinates.

Portion of the turbinate	Mean gland area (μm2)	Mean total area (μm2)	Gland quotient
Head	36976,684	296239,835	0,12485
Body	30379,222	293402,175	0,10381
Tail	31077,368 (p = 0,836)*	295036,679 (p = 0, 905)*	0,10567 (p = 0,836)*

* Applying Friedman's non-parametric test

## DISCUSSION

Generally in nasal surgery the intention is to achieve good ventilatory function without altering nasal physiology; major resection of the nasal mucosa, which might cause nasal drying and crusts or altered nasal airflow, is avoided. One of the main aims of surgical treatment is to balance the improvements in rhinorrhea and nasal obstruction with nasal drying.

Some authors have recommended submucosal turbinectomy in allergic rhinopathy patients, based on the idea that this technique preserves the respiratory epithelium and decreases the number of inflammatory cells in the nasal mucosa.^9^,^10^ Other authors^11^, ^12^, ^13^ are concerned with assessing the physiology and/or histopathology of the nasal mucosa before indicating turbinectomy. In other words, detailed histological knowledge of the mucosa of the lower nasal turbinates is essential for adequate surgical planning and improved results.

The site and quantity of seromucous glands and production of mucus are further concerns. Patients with vasomotor rhinopathy have fewer glands in the lamina propria, whereas allergic rhinopathy patients have relatively more of such glands; patients with nasal turbinate hypertrophy secondary to septal deviation have a normal number of seromucous glands.^12^

Thus, histological studies of each anatomical portion of the nasal turbinates are needed for planning surgery or medical therapy and for understanding the pathophysiology of nasal diseases.

This study may also be compared with other similar investigations that study the seromucous glands of the lower nasal turbinates. Many studies have assessed the histology and immunohistochemistry of the lower nasal turbinate mucosa to investigate vasomotor, allergic and atrophic rhinopathies, among others, which may be associated with gland activity.^15^,^16^ For instance Nakaya et al.^17^, ^18^ found muscarinic and histamine receptors in seromucous glands. Knowing the distribution of these glands in the lower nasal turbinates is relevant for performing biopsies in previously investigated areas (anterior, middle or posterior portions of the lower nasal turbinate). Standardization of studies would be improved, since data gathering would not be done randomly, but rather in areas in which the glands are known to be present or absent.

Also useful is to know in which areas these glands may be increased in size. Areas to be reached in patients with rhinorrhea can be established more effectively; similarly, conservative surgery may be done to avoid postoperative drying of the nose. Studying the distribution pattern of glands in the lower nasal turbinates is relevant for applying topical medication in the nose in the appropriate sites.

There are no histological studies in the literature that compare the various areas of the lower nasal turbinates.

Based on our results, seromucous glands in the lower nasal turbinates are distributed uniformly in the anterior, middle and posterior areas. Thus, there is no increased local concentration of glands. Similar studies should also be carried out in patients with rhinopathies to support specific therapies, whether applying medication in specific areas, or performing more conservative turbinectomies in specific areas of the nasal turbinates.

## CONCLUSION

This study shows that epithelial glands are not concentrated in any specific area of the lower nasal turbinates in the anterior to posterior direction in normal patients.
